# The effects of treading by two breeds of dairy cow with different live weights on soil physical properties, poaching damage and herbage production on a poorly drained clay-loam soil

**DOI:** 10.1017/S0021859614001099

**Published:** 2014-10-16

**Authors:** P. TUOHY, O. FENTON, N. M. HOLDEN, J. HUMPHREYS

**Affiliations:** 1 Animal and Grassland Research and Innovation Centre, Teagasc, Moorepark, Fermoy, Co. Cork, Ireland; 2 UCD School of Biosystems Engineering, University College Dublin, Belfield, Dublin 4, Ireland; 3 Environmental research centre, Teagasc, Johnstown Castle, Wexford, Co. Wexford, Ireland

## Abstract

There is little empirical evidence to indicate that dairy cow live weight affects the extent of soil damage at the hoof-soil interface during grazing on poorly drained permanent grassland. In the present study the impact of Holstein-Friesian (HF) dairy cows with a mean (±standard deviation) live weight of 570 (±61) kg were compared with Jersey × Holstein-Friesian (JX) with a mean live weight of 499 (±52) kg each at two stocking densities: mean 2·42 ± (0·062) and 2·66 (±0·079) cows/ha. Soil physical properties (bulk density, macroporosity, gravimetric water content, air-filled porosity, penetration resistance and shear strength), poaching damage (post-grazing soil surface deformation and hoof-print depth), herbage yield and milk production were measured throughout 2011 and 2012. Soil physical properties, post-grazing soil surface deformation and herbage production were not affected by dairy cow breed or by interactions between breed and stocking density. Hoof-print depth was higher in the HF treatments (39 *v.* 37 mm, s.e. 0·5 mm). Loading pressure imposed at the soil surface was the same for both breeds due to a direct correlation between live weight and hoof size. Poaching damage was greater at higher stocking density. Using the lighter JX cow offered little advantage in terms of lowering the negative impact of treading on soil physical properties or reducing poaching damage and no advantage in terms of herbage or milk production compared with the heavier HF cow.

## INTRODUCTION

Producing milk from grazed grass on poorly drained soils is an important aspect of agricultural production in temperate regions. Approximately 0·50 (3·4 m ha) of the total land area of Ireland is classified as ‘marginal’ and affected by natural limitations related to its soil, topography, relief and climate (Gardiner & Radford 1980). The principle limitation of this marginal land is its poor drainage status (Mulqueen 1974; Burdon 1986; Galvin 1987). It has been estimated that 0·46 of Irish farms are on land classified in the Teagasc National Farm Survey as ‘limited in agricultural use because of land wetness’ (Hennessy *et al*. 2011). Poorly drained soils typically remain wet for prolonged periods each year, reach saturation during rain events and remain wetter than field capacity for a number of days, even when no further precipitation occurs (Schulte *et al.* 2005). Farms on such soils have a shorter grazing season, lower proportion of grazed grass in the diet and consequently lower profitability when compared to farms on free-draining soils (Shalloo *et al.* 2004; Fitzgerald *et al.* 2008). The shorter grazing season is due to the necessity of keeping cows indoors to avoid excessive soil damage and by lower pasture production (Mullen *et al.* 1974; Patton *et al.* 2012).

Soil physical damage caused by cattle treading may involve a reduction in soil porosity with no indication of animal treading at the surface (Herbin *et al.* 2011). It can also involve a permanent displacement and remoulding of the soil surface around the hoof at higher water contents or a puddled soil under extremely wet conditions (Greenwood & McKenzie 2001; Drewry *et al.* 2008). Where soil and pasture are damaged there can be a loss in both utilization of the sward and in subsequent pasture production (Drewry *et al.* 2008; McDowell 2008).

Surface deformation coupled with compaction at depth results in increased soil resistance and reduced pore space (Betteridge *et al.* 1999; Batey & McKenzie 2006), which affects both shoot and root growth (Cook *et al.* 1996; Houlbrooke *et al.* 1997). Plant growth may also be reduced by direct effects of poaching including plant injury, fragmentation and burial (Brown & Evans 1973; Menneer *et al.* 2005; Phelan *et al*. 2013*a*). The resistance of soil to deformation is dependent on soil moisture (Mapfumo & Chanasyk 1998); however, intensively grazed grassland is often situated in regions with high rainfall (Smit *et al.* 2008). The extent of soil damage in poorly drained soils is dependent on factors that are fixed (soil type and climate) and non-fixed (animal live weight, stocking density and grazing duration). The non-fixed elements may be managed to overcome soil and pasture damage (Scholefield & Hall 1986; Finlayson *et al.* 2002).

Interest in crossbreeding Holstein-Friesians (HF) with Jersey cattle is fuelled by the inadequate reproductive performance of HF cows in pasture-based systems (Prendiville *et al.* 2009; Heins *et al.* 2012). The Jersey × HF (JX) cow is naturally lighter than the HF breed and is perceived to offer an advantage on poorly drained soils by imposing less treading damage during grazing (Herbin *et al.* 2011). This perceived advantage has not been investigated at a farm scale.

It is hypothesized that the use of lighter JX cows, relative to HF cows, reduces poaching damage and the adverse effects of treading during grazing on soil physical properties under typical grazing management. The objective of the current paper was to investigate the effects of dairy-cow breed (JX *v.* HF) on: soil surface loading pressures, soil physical properties [bulk density (BD), macroporosity (MP), gravimetric water content (GWC), air-filled porosity (AFP), penetration resistance (PR) and shear strength (SS)] and poaching damage [post-grazing soil surface deformation (SSD) and hoof-print depth (HPD)] in the upper layers of a clay-loam soil under locally typical grazing management practices on poorly drained soil. The effects on herbage production and on milk production were also examined. Total nitrogen (N) inputs and uptake are presented to provide a context for herbage production data.

## MATERIALS AND METHODS

### Site description

The present study was conducted at Solohead Research Farm (52 ha) in Ireland (52°30′N, 08°12′W, 95 m a.s.l.) during 2010, 2011 and 2012. Average annual rainfall over the previous 10 years was 1018 mm, with potential evapotranspiration of *c*. 510 mm. Soils are predominantly poorly drained Gleys (0·90 of the farm) and grey brown podzolics (0·10) of clay-loam texture with a perched water table, 0·0–2·2 m below ground level (Necpalova *et al.* 2012). The farm was drained in the 1960s and 1990s with deep open drains controlling water table depth. Nevertheless, much of the farm remains seasonally waterlogged. The farm has been under permanent grassland for at least 50 years.

### Experimental treatments and design

The experiment was set up during 2010. In spring 2010 an experimental area of 38·4 ha was divided into six blocks, each with uniform soil type and drainage status. Each block was divided into four paddocks. There were two breeds of dairy cow: (1) HF with an average live weight of 570 kg (s.d. = 61 kg), and (2) JX with an average live weight of 499 kg (s.d. = 52 kg). During 2010 two paddocks in each block were randomly assigned to HF and the other two to JX. Paddocks assigned to each breed treatment were rotationally grazed by herds of 48 cows of each breed during the 2010 grazing season (1 February to 16 December) and surplus herbage was removed as silage. The mean effective annualized stocking rate of both breeds during 2010 was 2·52 cows/ha. The milk yield capacity of both breeds was ascertained during 2010 and used to determine the stocking density treatments to be imposed during 2011 and 2012.

In 2011 and 2012, two stocking densities of each breed were imposed in a randomized block design with two factors and six replicated blocks. Annual stocking densities are presented in Table 1. In spring 2011 and 2012, all cows of each breed were divided into groups on the basis of lactation number (1, 2, 3 and ⩾4) and then sub-divided into sub-groups of two on the basis of calving date. From within each sub-group, one cow was randomly assigned to each stocking density treatment. The experiment consisted of 96 dairy cows (48 HF and 48 JX) in 2011 and 100 dairy cows (50 HF and 50 JX) in 2012. Mean calving date was February 28 (s.d. = 26 days).The four herds were identified as HF-low stocking density (HF-L), HF-high stocking density (HF-H), JX-low stocking density (JX-L) and JX-high stocking density (JX-H). Herds were assigned to paddocks each spring. Paddocks assigned to HF were grazed only by HF during 2010, 2011 and 2012 and likewise for JX. Paddocks grazed by HF-L in 2011 were again grazed by HF-L in 2012 and likewise for the other treatments.

**Table 1.** Details of treatments imposed during the experiment. Dairy cow breeds were Holstein-Friesian and Jersey × Holstein-Friesian and annual fertilizer N input was 110 kg/ha supplemented by biologically fixed N from clover in the sward (Low) and 280 kg/ha (High)

Year	2011				2012			
Treatment herd	HF-L	HF-H	JX-L	JX-H	HF-L	HF-H	JX-L	JX-H
Mean cow live weight (kg)	573	561	487	475	572	573	515	519
Start of grazing season	18 Feb				7 Feb			
End of grazing season	17 Nov				17 Nov			
Land area (ha)	10·2	9·38	10·06	9·09	10·2	9·38	10·06	9·09
Annual stocking density (cows/ha)	2·35	2·56	2·39	2·64	2·45	2·67	2·49	2·75
Mean days grazing/cow*	231	230	233	233	196	194	200	201
Monthly stocking-density on the proportion of the farm used for grazing (cows/ha)								
Feb–Mar	2·35	2·56	2·39	2·64	2·45	2·67	2·49	2·75
Apr–mid-Jun	9·60	8·10	6·42	9·20	6·16	5·42	5·20	5·49
Mid-Jun–mid-Jul	4·67	6·63	4·87	7·77	2·45	2·95	2·49	3·49
Mid-Jul–Nov	2·35	2·56	2·39	2·64	2·45	2·67	2·49	2·75
Mean	4·38	4·69	3·82	5·23	3·19	3·29	3·03	3·48
Proportion of area used for silage production								
1st cut (Apr-mid Jun)	0·75	0·68	0·63	0·71	0·60	0·51	0·52	0·50
2nd cut (Mid Jun–mid Aug)	0·50	0·61	0·51	0·66	0·00	0·10	0·00	0·21
Intake per cow (kg DM/cow)								
Grazed pasture	3240	3256	3008	3197	2649	2376	2445	2552
Silage	1474	1485	1452	1452	1859	1881	1815	1804
Concentrate	496	495	495	495	873	867	837	842
Total DM intake	5210	5236	4955	5144	5381	5124	5097	5198
Clover production								
Proportion of white clover in herbage DM	0·282	0·259	0·274	0·268	0·167	0·132	0·168	0·148
White clover herbage yield (kg DM/ha)	3756	3824	3759	4034	1854	1512	1962	1721
Nitrogen inputs (kg/ha)								
Fertilizer	110	280	110	280	110	280	110	280
Slurry	56	56	56	56	57	57	57	57
Biologically fixed N	115	36	115	38	57	14	60	16
N deposited by cows	280	301	255	273	281	275	258	267
Total	561	673	536	646	505	627	485	620
Nitrogen uptake(kg/ha)	489	560	492	516	407	435	419	400
Nitrogen excess(kg/ha)	72	113	44	130	98	191	66	220

HF-L, Holstein-Friesian at low stocking density; HF-H, Holstein-Friesian at high stocking density; JX-L, Jersey × Holstein-Friesian at low stocking density; JX-H, Jersey × Holstein-Friesian at high stocking density

* 24-h period: when cows were housed at night a value of 0·5 was assigned

#### Grazing management

Cows were turned out to graze 3 days after calving from early February and remained at pasture until they were housed full-time, typically at the end of November. Exceptions were made when ground conditions were too wet (soil moisture >0·60 m^3^/m^3^) or when herbage supply was too low, which generally occurred when herbage growth rates were below demand and pre-grazing herbage mass was <500 kg dry matter (DM)/ha (above post-grazing height of 40 mm). On such occasions, all cows were housed. Strip grazing with temporary fencing was practised in all systems. Each herd was moved to the next strip when a post-grazing sward height [measured with a Filips rising plate meter (Grasstec, Mallow, Cork, Ireland)] of *c*. 40 mm was reached. Back fencing was used to stop cows returning to previously grazed areas.

Excess herbage was identified throughout the experiment and removed for silage production. These areas were selected when herbage growth rates exceeded demand, resulting in pre-grazing herbage mass >2000 kg of DM/ha (above post-grazing height of 40 mm). The entire area of each system was available for grazing during the spring and autumn. Silage areas were closed off between early-April and mid-June (first-cut silage) or between mid-June and mid-July (second-cut silage). The land area available for grazing in each system at various times of the year and associated stocking densities are outlined in Table 1.

Annual fertilizer N input was 110 kg/ha on the low stocking-density paddocks (HF-L and JX-L), which also relied on biologically fixed N facilitated by white clover in the sward, and 280 kg/ha on the high stocking-density systems (HF-H and JX-H), to cater for the increased grazing demand (Humphreys *et al.* 2008). Fertilizer N was applied in the form of urea between February and April and as calcium ammonium nitrate from May to September. Slurry produced during housing was stored and applied back equally to each treatment (Table 1).

#### Concentrates and silage fed

Cows received concentrate feed (0·26 barley, 0·26 maize gluten, 0·35 beet pulp and 0·12 soybean meal) at rates of 3–5 kg/cow/day from February to April and between 0 and 4 kg/cow/day from April to November, depending on herbage availability and nutritive value. When housed, cows were fed grass-clover silage *ad libitum*.

### Experimental measurements

#### Meteorological data

Weather data [temperature (°C), relative humidity (g/kg), rainfall (mm), wind speed (m/s) and direction (°) and solar radiation (J/m^2^)] were measured and recorded on site by an automated weather station (Campbell Scientific Ltd., Loughborough, UK) every 15 min. Daily data collected (rainfall, maximum and minimum air temperature, wind speed and solar radiation) were used as inputs to the model of Schulte *et al.* (2005) to estimate daily evaporation, effective drainage and soil moisture deficit (SMD).

#### Herbage production and white clover content

Exclusion plots were set up in four paddocks of each treatment. Each plot was 11 × 2·5 m and was positioned centrally in each paddock. Cows were prevented from walking or grazing in the plot by electrified wire. Exclusion plots were moved three times each year (February, May and July) to an adjacent area in the paddock, which had been subject to treading on previous grazing rotations to take account of the effect of treading on herbage yields.

Herbage yield was measured by cutting a 10 × 1·2 m strip in each exclusion plot prior to each grazing or silage cut using an Etesia Hydro 124DS Lawnmower (Etesia UK Ltd., Shenington, Oxon, UK) at a cutting height of 40 mm above ground level. The harvested herbage was weighed to determine herbage mass. A 100 g sub-sample was collected and then dried in a force-draught oven for 16 h at 100 °C to determine DM content. Annual herbage yield (kg of DM/ha) was calculated as the sum of herbage removed as pre-grazing and pre-silage cuts.

A second 100-g sub-sample of herbage was freeze-dried and milled through a 0·2 mm sieve before analyses for ash content (550 °C muffle furnace for 12 h), crude protein (CP; N content; Leco 528 auto-analyser, Leco Corp., St. Joseph, MI, USA) and *in vitro* organic matter digestibility (OMD) as described by Morgan *et al.* (1989). Silage fed was sampled throughout the experiment by taking grab samples of 100 g before feeding. These were analysed for ash, OMD and CP using near-infrared spectroscopy (model 6500, Foss-NIR System, Hillerod, Denmark).

Pasture cover was measured on a weekly basis in all paddocks to estimate weekly herbage growth using a Filips rising plate meter (Grasstec, Mallow, Cork, Ireland). Each week 40–60 compressed sward height (CSH) measurements were taken diagonally across each paddock. The average herbage mass above a cutting height of 40 mm was then calculated according to the following formula: Herbage mass = [Mean CSH (mm)−40 mm] × sward density; kg DM/mm/ha, assuming a sward density of 24 kg DM/mm/ha. Annual herbage production was calculated as the sum of weekly herbage growth throughout the year.

The proportion of white clover (*Trifolium repens* L.) in herbage in each paddock was measured using the methodology described by Phelan *et al.* (2013*b*).

#### Total nitrogen input and uptake

Total N input to the treatments was made up of fertilizer N, N applied in slurry, biologically fixed N from white clover and N deposited by grazing cows. The biologically fixed N in stolons, roots and stubble in each paddock was estimated from white clover content in the sward and herbage production using a mechanistic model as described by Humphreys *et al.* (2008). Nitrogen excretion by the dairy cows was estimated as the difference between intake of N and N deposited in milk, in calves or in live weight change of the cows and ammonia losses (Humphreys *et al.* 2008). Nitrogen uptake was calculated from the mean annual N content of harvested herbage in each system and its respective annual herbage yield.

#### Length of the grazing season, concentrates fed and intake estimate

The length of the grazing season was measured in terms of days at pasture where 1 day was defined as when all the cows per system were out day and night and one-half day when cows were out only by day. In spring when lactating cows were outdoors and cows yet to calve remained indoors, the proportion of cows outdoors was recorded. Likewise, when cows were being housed as they were dried off in the early winter the proportion of cows outdoors per system was recorded.

The amount of concentrate fed per cow was recorded at each milking (Dairymaster, Causeway, Co. Kerry, Ireland) and silage intake during housing was estimated as silage fed to cows minus rejected material. Intake of grazed pasture DM by each cow was estimated as the difference between net energy (NE) provided from silage and concentrate and that needed to meet the NE requirements for milk production, maintenance and pregnancy (Jarrige *et al.* 1986; Jarrige 1989; O'Mara 1996).

#### Milk yield and composition

Milking was conducted at 07·30 h each morning and 15·30 h each evening. Milk yield per cow (kg) was recorded at each milking and milk composition (fat, protein and lactose concentrations) from each cow was measured twice weekly on a successive morning and evening milking using a Milkoscan 203 (Foss Electric DK-3400, Hillerød, Denmark). Solids-corrected milk yield was calculated using the equation of Tyrell & Reid (1965).

#### Soil properties

Soil samples were collected using a soil sampling ring kit and stainless steel cores having a volume of 9·82 × 10^−5^ m^2^ (Eijkelkamp, Agrisearch Equipment, Giesbeek, The Netherlands), from the layer at 0·05–0·10 m below ground level and at 0·15–0·20 m below ground level. Nine locations per paddock were selected at random in each of four paddocks per treatment within four replicate blocks at the start of the experiment (February 2011). The global positioning system (GPS) coordinates of each location was recorded. Soil cores at each depth were collected on four occasions (February, May, August and November) each year.

Soil BD (mass of soil per unit volume, kg/m^3^), MP [proportion of soil volume occupied by pore spaces >30 μm in diameter (Drewry *et al.* 2008), m^3^/m^3^], gravimetric water content (GWC, mass of water per mass of soil, g/g) and AFP (proportion of pore space occupied by air, m^3^/m^3^) were measured using standard methods (Piwowarczyk *et al.* 2011; Phelan *et al.* 2013*a*)

A plasticity index test (British Standards Institute 1990) to determine Atterberg limits was conducted on two bulk soil samples (Metlab Ltd, Ballygarvan, Cork, Ireland). One sample was collected from each of two depths, 0·00–0·10 m and 0·10–0·20 m, at a randomly selected location in each paddock in March 2012; samples were then bulked for each depth.

#### Soil strength

Soil PR (resistance of the soil to penetration by a cone, per unit area of the cone's base, in Mpa) was measured using a penetrologger (Eijkelkamp, Agrisearch Equipment, Giesbeek, The Netherlands). Shear strength (the magnitude of shearing stress that a soil can sustain in kPa) was measured using a standard shear vane (Eijkelkamp, Agrisearch Equipment, Giesbeek, The Netherlands). Ten measurements (Bengough *et al*. 2001) of each were taken in the vicinity of the locations described above in four paddocks per treatment within four replicate blocks on four occasions annually (January, April, July and October).

The PR was measured to a depth of 0·30 m below ground level and averaged over 0.05 m intervals to a depth of 0·30 m for each treatment for analysis. These measurements will be referred to as PR5 (mean PR at 0·00–0·05 m depth), PR10 (mean PR at 0·06–0·10 m depth) and so on. The SS was determined at depths of 0·05, 0·10 and 0·15 m below ground level.

The soil volumetric moisture content (SVMC) was measured simultaneously with PR and SS measurements: SVMC in the upper 0·05 m of soil was measured using an ML2× soil moisture measurement kit (Delta-T Devices Ltd, Burwell, Cambridge, UK) at 20 locations in the vicinity of the PR and SS measurements.

#### Poaching damage

Soil surface deformation (m/m) provided a standardized quantitative method of determining the rate of poaching damage. It was measured by fitting a 2-m long chain to the soil surface, taking account of any changes in micro-topography. The profile length of the chain was measured against a 2-m long wooden staff. Deformation was quantified as the reduction in the chain's profile length relative to the staff (Saleh 1994; Nie *et al.* 2001; Phelan *et al.* 2013*a*). Hoof-print depth (mm) was measured with callipers. Soil surface deformation and HPD were recorded immediately after each grazing in each paddock. On each occasion two locations were selected at random in the most recently grazed section of the paddock. At each location two SSD measurements and ten HPDs were recorded. The SVMC was recorded simultaneously with SSD and HPD; ten measurements were taken at each location using the ML2×  soil moisture measurement kit as described above.

#### Cow live weight and body condition score

The live weight of each cow was recorded fortnightly using weighing scales and the Winweigh software package (Tru-Test Limited, Auckland, New Zealand). Body condition score (Edmonson *et al.* 1989) of each dairy cow was recorded once a fortnight.

#### Hoof surface area and surface loading pressure

In October 2012, all cows of each breed were divided into four main groups on the basis of lactation number (1, 2, 3 and ⩾4). From within each group, 0·4 of the total number of cows were selected at random for hoof size measurement. The surface area of the back left hoof of this subset (*n* = 20 for each breed) was measured and taken to represent the average surface area of hooves of cows in each treatment. Static surface loading pressure (kPa) was calculated as the mass of the cow (kg) divided by the total hoof area (m^2^), multiplied by 100.

#### Statistical analysis

Cow hoof size and live weight were analysed as a single factor (breed) analysis of variance (ANOVA) having 48 or 50 replicates. Static loading pressure was analysed by ANOVA with only breed as a factor with 20 replicates. All other animal production data (milk production, milk composition, cow live weight, body condition score, diet and management) were analysed as a three factor (year × breed × stocking density) ANOVA with 24 or 25 replicates. Herbage production data were analysed as a three factor (year × breed × fertilizer regime) ANOVA, with four replicates for the method using exclusion plots and six replicates for the method using growth rates. Clover content was analysed as a four factor (year × season × breed × fertilizer regime) ANOVA with six replicates.

Soil data (BD, MP, GWC, AFP, PR and SS) were analysed as two factor (breed × stocking density) ANOVA examining the main effects of factors and interactions between factors, with four replicates. The SSD and HPD data were analysed by ANOVA including year, breed and stocking density, with four replicates. The significance of any correlations within the results was analysed with linear regression through the analysis of covariance in the PROC MIXED procedure in SAS version 9.1.3 (SAS Institute 2006).

## RESULTS

### Rainfall and soil moisture deficit

**Fig. 1.**
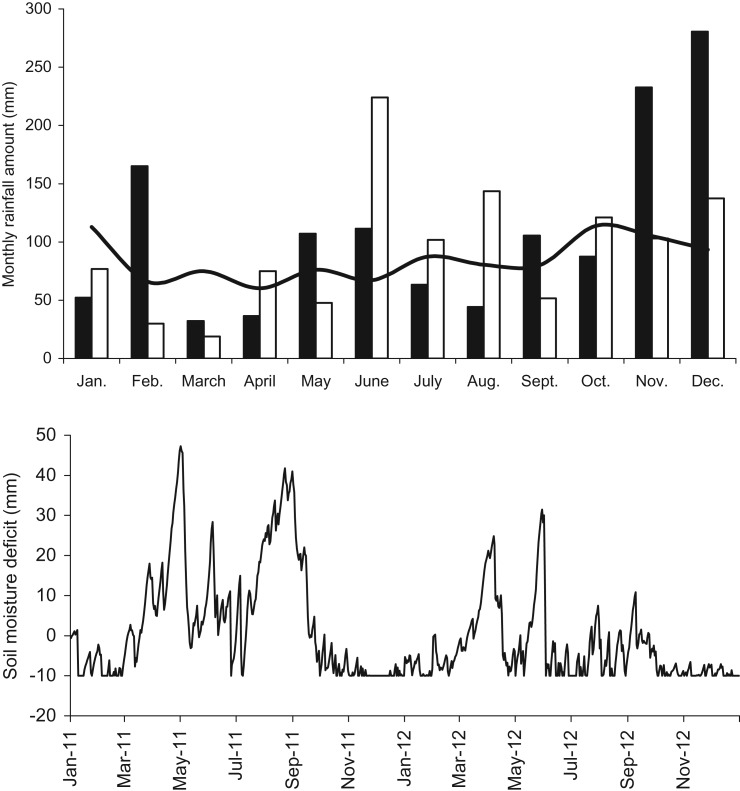
(*a*) Monthly rainfall (mm/month) recorded in 2011(■) and 2012 (□) at the meteorological station at Solohead Research Farm. The solid black line shows the previous 10-year mean values and (*b*) SMD (mm/day) estimated from data recorded at the meteorological station at Solohead Research Farm for the study period.

Annual rainfall was 1318 mm in 2011 and 1131 mm in 2012. In both years rainfall was above the mean of the previous 10-year period (1018 mm; range 797–1296 mm), (Fig. 1(*a*)). In 2011 the three wettest months were February, November and December. Both November and December 2011 each had higher monthly rainfall than that recorded in any month in the previous 10 years. These three months accounted for 678 mm of rainfall (or 0·51 of annual rainfall in 2011). During the period 1 March to 31 October 2011, rainfall was 588 mm compared to the 10-year average of 640 mm. In 2012, rainfall was concentrated in the summer months with double the 10-year average for the 3-month period encompassing June, July and August; 469 *v.* 235 mm. During the period 1 March to 31 October 2012 rainfall was 784 mm.

Mean annual SMD was 4·5 mm/day in 2011 and –3·9 mm/day in 2012. Saturated soil conditions (SMD = –10 mm) occurred on 73 days in 2011 and 88 days in 2012 while there was excess soil water above field capacity (SMD < 0 mm) on 179 days in 2011 and 285 days in 2012 (Fig. 1(*b*)).

### Herbage production and white clover content

Herbage production was not significantly affected by breed or interactions between breed and the other factors. Herbage production was greater (*P* < 0·01) at higher N input. Annual herbage yield was higher (*P* < 0·001) in 2011 than 2012 (14 334 *v.* 11 954 kg/ha, s.e. 285·5 kg/ha, Table 2). Clover content in herbage DM was not affected by breed, N input or interactions with breed or N input. Clover content in herbage DM was higher (*P* < 0·001) in 2011 than 2012 (0·271 *v.* 0·154, s.e. 0·0094). Mean clover herbage DM yield across systems was 3843 kg/ha in 2011 and 1762 kg/ha in 2012 (Table 1).

**Table 2.** The effect of dairy cow breed (Holstein-Friesian and Jersey × Holstein-Friesian), annual fertilizer N input (low = 110 kg/ha supplemented by biologically fixed N from clover in the sward, high = 280 kg/ha) year, on annual herbage production (kg/ha) by two methods

	Year	Dairy cow breed		Fertilizer N input		s.e.m.						
Method		HF	JX	Low	High	Year	Breed	N input	Year × breed	Year × N input	Breed × N input	Year × breed × N input
Exclusion plot cut yields	2011	14 263	14 406	13 813	14 856	286	286	286	404	404	404	571
	2012	12 027	11 882	11 785	12 123	(*P* < 0·001)	NS	(*P* < 0·01)	NS	NS	NS	NS
Weekly pasture growth	2011	13 830	14 360	13 214	14 976	335	335	335	474	474	474	670
	2012	10 589	11 440	10 982	11 017	(*P* < 0·001)	NS	(*P* < 0·01)	NS	(*P* < 0·05)	NS	NS

HF, Holstein-Friesian; JX, Jersey × Holstein-Friesian; N, nitrogen; NS, not significant

### Total nitrogen input and uptake

Total N input (including fertilizer, N applied in slurry, biologically fixed N and N deposited by grazing cows) in the low fertilizer N treatments was 549 and 494 kg/ha in 2011 and 2012, respectively. In the high fertilizer N treatments total N input was 662 and 625 kg/ha in 2011 and 2012, respectively. The excesses of inputs over uptake were higher in the high fertilizer N treatments (121 and 206 kg/ha in 2011 and 2012, respectively) relative to the low fertilizer N treatments (58 and 82 kg/ha in 2011 and 2012, respectively) (Table 1).

### Length of the grazing season, concentrates fed and intake estimate

The total number of days grazing in each system is shown in Table 1. The JX had an earlier mean calving date than the HF. This impacted significantly (*P* < 0·01, s.e. 0·91 days) on the number of grazing days with 212·5 days/cow for HF and 216·8 days/cow for JX. The number of grazing days was not affected by stocking density treatment. The grazing season was longer (*P* < 0·001) in 2011 than in 2012 (232 *v.* 198 days/cow; s.e. 0·91). The amount of concentrates fed per cow did not differ between breeds or between stocking density treatments. On average cows received 495 kg of concentrates in 2011 and 855 kg in 2012 (*P* < 0·001, s.e. 6·0 kg). Table 1 presents estimates of grazed pasture and silage intake and measured concentrate intakes per cow (kg DM/cow).

### Milk yield and composition

Annual milk yield per cow was significantly higher (*P* < 0·001) for the HF breed (5841 *v.* 5504 kg, s.e. 68·0 kg), but JX had higher fat (50·7 *v.* 46·3 g/kg, s.e. 0·53 g/kg) and protein (both *P* < 0·001, 38·7 *v.* 36·3 g/kg, s.e. 0·29 g/kg) and higher lactose (*P* < 0·05, 47·8 *v.* 46·9 g/kg, s.e. 0·27 g/kg) concentration than HF. There was no difference between breeds in annual production per cow of yields of solids-corrected milk, milk fat and milk protein. The HF cows produced higher yields of lactose (*P* < 0·05, 274 *v.* 263 kg, s.e. 3·6 kg).

Stocking density did not affect milk production per cow. The higher stocking density treatments yielded more milk (15 227 *v.* 13567 kg/ha), solids-corrected milk (16 181 *v.* 14737 kg/ha), milk fat (725 *v.* 664 kg/ha), milk protein (564 *v.* 513 kg/ha) and milk lactose (717 *v.* 646 kg/ha) on a per hectare basis.

### Soil properties

Soil properties BD, MP, GWC and AFP at the 0·05–0·10 m depth range were not affected by breed, stocking density or interaction between breed and stocking density. All soil properties measured showed strong correlations with GWC across treatments. Mean AFP at 0·05–0·10 m depth across treatments was 0·17 m^3^/m^3^ (ranging from 0·11 to 0·23 m^3^/m^3^) in 2011 and 0·12 m^3^/m^3^ (ranging from 0·09 to 0·17 m^3^/m^3^) in 2012. Measurements taken at the 0·15–0·20 m depth range showed similar responses in BD, MP, GWC and AFP as those at 0·05–0·10 m. The treatments imposed did not have an effect on these soil properties at the 0·15–0·20 m depth range.

### Soil strength

Penetration resistance at all depths up to 0·25 m was not affected by cow breed, interaction between breed and stocking density, or stocking density. The PR30 was not affected by breed but was higher (*P* < 0·05) at the low stocking density than the high stocking density (2·51 *v.* 2·05 Mpa, s.e. 0·117 Mpa). Breed, interaction between breed and stocking density or stocking density had no effect on soil SS at any depth.

### Poaching damage

**Fig. 2.**
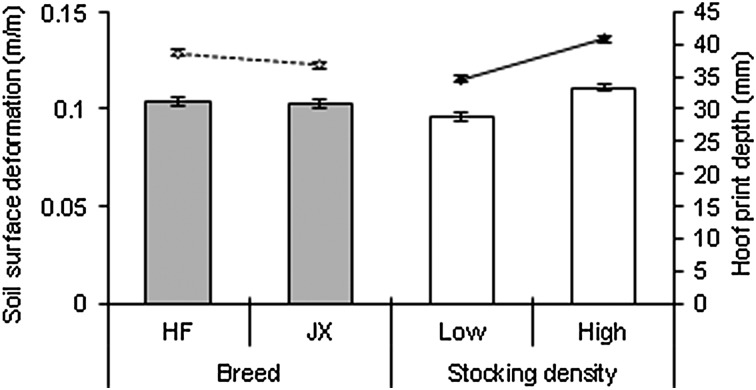
Effect of breed, on SSD (grey columns, no significant difference) and HPD (dashed line, *P* < 0·01), and effect of stocking density on SSD (white columns, *P* < 0·001) and HPD (solid line, *P* < 0·001), error bars show the treatment s.e.m.

Hoof-print depth (Fig. 2) was greater under the HF cows (*P* < 0·01, 39 *v.* 37 mm, s.e. 0·5 mm) and was affected significantly (*P* < 0·001) by the interaction between year, breed and stocking density (Table 3). Hoof-print depth was higher (*P* < 0·001) at the higher stocking density (41 *v.* 35 mm, s.e. 0·5 mm) and was affected (*P* < 0·01) by an interaction between year and stocking density. Stocking density had an effect on HPD in both years, but this was more pronounced in 2012 (Table 3). Soil surface deformation was not affected by breed but was affected (*P* < 0·05) by the interaction between year, breed and stocking density. Soil surface deformation (Fig. 2) was greater at higher stocking density (*P* < 0·001, 0·11 *v.* 0·10 m/m, s.e. 0·0020 m/m) and was affected (*P* < 0·05) by an interaction between year and stocking density: Stocking density affected SSD in both years, but more so in 2012 (Table 3).

**Table 3.** The effect of dairy cow breed (Holstein-Friesian and Jersey × Holstein-Friesian), at two stocking densities (low; 2·42 cows/ha and high; 2·66 cows/ha) and year on HPD (mm) and SSD (m/m)

	Dairy cow breed				Stocking density				s.e.m.						
	2011		2012		2011		2012								
	HF	JX	HF	JX	L	H	L	H	Year	Breed	Stocking density	Year × breed	Year × stocking density	Breed × stocking density	Year × breed × stocking density
Hoof-print depth (mm)	29	27	49	46	26	30	43	52	0·5 (*P* < 0·001)	0·5 (*P* < 0·01)	0·5 (*P* < 0·001)	0·7 NS	0·7 (*P* < 0·01)	0·7 NS	1·1 (*P* < 0·001)
Soil surface deformation (m/m)	0·07	0·07	0·14	0·14	0·06	0·07	0·13	0·15	0·002 (*P* < 0·001)	0·002 NS	0·002 (*P* < 0·001)	0·003 NS	0·003 (*P* < 0·05)	0·003 NS	0·004 (*P* < 0·01)

HF, Holstein-Friesian; JX, Jersey × Holstein-Friesian; L, low stocking density; H, high stocking density; NS, not significant

Soil surface deformation was significantly correlated with HPD (*y* = 2·46*x* + 12·29, *R*^2^ = 0·75, *P* < 0·001). Mean SSD was lower (*P* < 0·001) in 2011 (mean ±  s.d.), (0·07 ± 0·042 m/m) compared with 2012 (0·14 ± 0·056 m/m). Mean HPD was lower (*P* < 0·001) in 2011 (28 ± 11·9 mm) than 2012 (48 ± 17·1 mm).

### Cow live weight, body condition score and hoof surface area

**Fig. 3.**
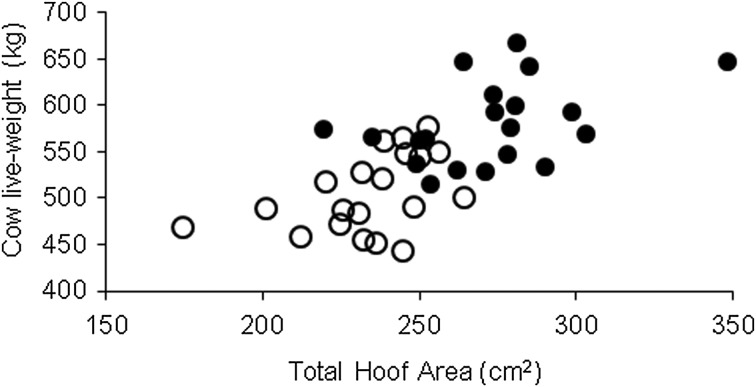
Relationship between cow live weight and total hoof area for HF (•) and JX (○) cows. HF cows are heavier (*P* < 0·001) and have larger hoofs (*P* < 0·001) than their JX equivalents.

The HF cows were heavier (*P* < 0·001) than JX: 570 *v.* 499 kg/cow, s.e. 4·0. Cows were heavier (*P* < 0·001, s.e. 4·0 kg) in 2012 (545) than 2011 (524 kg). The HF cows had a higher BCS than the JX cows, 3·13 compared with 3·05 (*P* < 0·001, s.e. 0·014). For the subset of 20 cows per treatment used for the calculation of surface loading pressure, HF were heavier (580 *v.* 506 kg, s.e. 6·6 kg, *P* < 0·001) than their JX equivalents while also having larger hooves (0·027 *v.* 0·023 m^2^, s.e. 0·0005 m^2^, *P* < 0·001). Hoof surface area was significantly correlated with cow live weight (*y* = 1·22*x* + 233·47, *R*^2^ = 0·45, *P* < 0·001; Fig. 3). There was no difference in surface loading pressure on the soil between the breeds (Table 4).

**Table 4.** Mean weight, total hoof area and static pressure of subset of Holstein-Friesian and Jersey × Holstein-Friesian cows measured from the herd

Breed	Mass (kg)	Total hoof area (m^2^)	Static pressure (kPa)
HF	580	0·027	214
JX	506	0·023	218
s.e.	6·6	0·0005	4·2
*P*	<0·001	<0·001	NS

HF, Holstein-Friesian; JX, Jersey × Holstein-Friesian; NS, not significant

## DISCUSSION

### Rainfall, nitrogen input and herbage production

Rainfall was above average in both years of the study. However, due to the rainfall patterns observed, ground conditions were drier than normal during the main grazing season in 2011 and substantially wetter than average in 2012.

Total N input estimates show that N available in different treatments was not solely dependent on the N fertilizer application rate. Those paddocks receiving less fertilizer N were augmented by biologically fixed N from white clover in the sward. Biologically fixed N was estimated to have contributed an extra 78 kg/ha in 2011 and 44 kg/ha in 2012 to the low fertilizer N systems. The higher N excesses in the high fertilizer N treatments relative to the low fertilizer N treatments indicate that although there was more N available in the high fertilizer N treatments a lower proportion was utilized for herbage production when compared to the low fertilizer N treatments. A similar result was attributed to low efficiency of fertilizer N use under high rainfall, probably due to high rates of denitrification (Humphreys *et al.* 2008).

Herbage production and the length of the grazing season were dependent on N input, weather and soil conditions. Due to excessively wet soils and poor herbage growth rates, cows were housed frequently during the 2012 grazing season. The total length of the grazing season was much less than in 2011, when housing during the grazing season was not necessary. Consequently, due to the lower amount of grazed grass in the diet, there was higher demand for silage and concentrate in all systems in 2012 than in 2011.

Poor conditions for herbage production prevailed in 2012. Optimum AFP for pasture production is in the range of 0·15 and 0·20 m^3^/m^3^ (Drewry *et al.* 2008); with a critical minimum value of 0·10–0·12 m^3^/m^3^ required to ensure adequate diffusion of air to plant roots (Lipiec & Hatano 2003). During 2012, mean AFP at 0·05–0·10 m depth across treatments was only in the optimum range in May at 0·17 m^3^/m^3^. Soil samples collected in February, August and November 2012 had mean AFP ranging from 0·09 to 0·11 m^3^/m^3^. It is assumed that air movement to the plant roots was restricted at these levels, thus restricting herbage production. In 2011, AFP was higher throughout the grazing season and only fell below the optimum level in November.

### Milk yield and composition

The results show no difference in yield of solids-corrected milk, fat and protein per cow between breeds. Similar comparisons between Holstein-Friesian and Jersey × Holstein-Friesian cows concur with these findings (Prendiville *et al*. 2009; Xue *et al.* 2011; Vance *et al.* 2013).

### Effect of treading on poaching damage

While there was no effect of breed on SSD and only a slightly higher HPD imposed by HF cows, there was a clear difference in poaching damage between the stocking density treatments, particularly in 2012. This difference is due to the effect repeated loading had on soil strength. A higher stocking density resulted in a greater frequency of hoof-soil interactions and a higher amount of surface damage, which was further exacerbated by wet conditions. The repeated treading of wet soil increases the depth to which the soil is weakened (Mullins & Fraser 1980). Thus partly poached soil is more susceptible to subsequent poaching as it is softer and wetter (Gradwell 1968). This process may also account for some of the difference in HPD recorded between breeds during the experiment. Since an HF hoof is 1·17 times larger than a JX hoof, HF treaded on 0·17 more of the pasture area than JX at each grazing. While the loading pressure is the same, the intensity of loading is increased, in much the same way as the intensity of hoof-soil interactions is increased with higher stocking rate.

The higher poaching damage in 2012 was due to the soil being above its plastic limit throughout the grazing season. The plastic limit of a soil is the GWC at which a soil changes from being friable to being plastic, and represents the lowest water content at which pugging and poaching may occur (Drewry *et al.* 2008). Analysis of the soil at 0·00–0·10 m depth, found the plastic limit was 0·43 g/g GWC. In 2011 this threshold was not breached until November when mean GWC was 0·51 g/g. Soil samples taken in 2012 show soil GWC was continually well above the plastic limit, ranging from 0·56 (August) to 0·68 (November) g/g. In this scenario soil deformation was the dominant effect of treading during grazing.

### Soil surface loading characteristics of dairy cattle

The soil surface loading pressure of a grazing animal is a function of the live weight and hoof size as well as the loading pattern during ambulation. The lowest magnitude of loading is applied by a stationary animal and is taken as the total live weight divided by the total contact hoof area. Greater pressures are evident when the animal is walking or running as not all hooves are in continuous contact with the soil surface and as such the live weight is distributed over a smaller contact area (Willatt & Pullar 1984; Greenwood & McKenzie 2001). For practical purposes it is assumed that the dynamic load of a moving cow is approximately twice that of an equivalent stationery cow (Scholefield & Hall 1985; Piwowarczyk *et al.* 2011).

It has been shown that no differences exist between the breeds used in the present study in terms of grazing time, number of grazing bouts and grazing bout duration in an intensive, seasonal grass-based system (Prendiville *et al.* 2010). Hence, the principle difference between the breeds was in live weight. The hoof size measurement methodology allowed for the likely increase in size with increasing age (Hahn *et al.* 1984; Boelling & Pollott 1998) by selecting a representative group on the basis of lactation number. Both breeds exhibited similar static pressures and, hence, similar dynamic pressures. While it is hypothesized that the lighter cow has less potential to cause damage, it has been shown that pressure applied is the same regardless of live weight. Surface loading pressure, not live weight, was the factor most affecting the soils response to treading.

## CONCLUSIONS

In general post-grazing soil physical properties and poaching damage were not influenced by dairy-cow breed, with only HPD being significantly affected by breed. Herbage production and milk production in terms of yield of milk fat and protein were not different between breeds. Therefore there was no advantage, in terms of lowering the negative impact of treading on soil physical properties or reducing poaching damage, in using the lighter JX cow rather than the heavier HF cow as a tactic to optimize production from poorly drained grassland soils. Lack of differences between the breeds was due to the correlation between body weight and hoof size, which was common to both breeds, hence the static pressure exerted at the soil surface was the same regardless of breed.

## References

[ref1] Batey T. & McKenzie D. C. (2006). Soil compaction: identification directly in the field. Soil Use and Management 22, 123–131.

[ref2] Bengough A. G., Campbell D. J. & O'Sullivan M. F. (2001). Penetrometer techniques in relation to soil compaction and root growth In Soil and Environmental Analysis: Physical Methods, 2nd edn (Eds K. A. Smith & C. E. Mullins), pp. 377–403. New York: Marcel Dekker.

[ref3] Betteridge K., Mackay A. D., Shepherd T. G., Barker D. J., Budding P. J., Devantier B. P. & Costall D. A. (1999). Effect of cattle and sheep treading on surface configuration of a sedimentary hill soil. Australian Journal of Soil Research 37, 743–760.

[ref4] Boelling D. & Pollott G. E. (1998). Locomotion, lameness, hoof and leg traits in cattle I: phenotypic influences and relationships. Livestock Production Science 54, 193–203.

[ref5] British Standards Institute (1990). BS 1377: Part 2: Methods of Test for Soils for Civil Engineering Purposes. Classification Tests. London: British Standards Institute.

[ref6] Brown K. R. & Evans P. S. (1973). Animal treading a review of the work of the late D. B. Edmond. New Zealand Journal of Experimental Agriculture 1, 217–226.

[ref7] Burdon D. J. (1986). Hydrogeological aspects of agricultural drainage in Ireland. Environmental Geology and Water Sciences 9, 41–65.

[ref8] Cook A., Marriott C. A., Seel W. & Mullins C. E. (1996). Effects of soil mechanical impedance on root and shoot growth of *Lolium perenne* L., *Agrostis capillaris* and *Trifolium repens* L. Journal of Experimental Botany 47, 1075–1084.

[ref9] Drewry J. J., Cameron K. C. & Buchan G. D. (2008). Pasture yield and soil physical property responses to soil compaction from treading and grazing – a review. Australian Journal of Soil Research 46, 237–256.

[ref10] Edmonson A. J., Lean I. J., Weaver L. D., Farver T. & Webster G. (1989). A body condition scoring chart for Holstein dairy cows. Journal of Dairy Science 72, 68–78.

[ref11] Finlayson J. D., Betteridge K., MacKay A., Thorrold B., Singleton P. & Costall D. A. (2002). A simulation model of the effects of cattle treading on pasture production on North Island, New Zealand, hill land. New Zealand Journal of Agricultural Research 45, 255–272.

[ref12] Fitzgerald J. B., Brereton A. J. & Holden N. M. (2008). Simulation of the influence of poor soil drainage on grass-based dairy production systems in Ireland. Grass and Forage Science 63, 380–389.

[ref13] Galvin L. F. (1987). Aspects of land drainage development in Ireland in the last twenty-five years. In *Proceedings, Symposium 25th International Course on Land Drainage: Twenty-Five years of Drainage Experience* (Ed. J. Vos), pp. 131–140. Publication 42. Wageningen, The Netherlands: International Institute for Land Reclamation and Improvement.

[ref14] Gardiner M. J. & Radford T. (1980). Soil Associations of Ireland and their Land Use Potential. Dublin: An Foras Taluntais.

[ref15] Gradwell M. W. (1968). Compaction of pasture topsoils under winter grazing. In *Proceedings of the Transactions of the 9th International Congress of Soil Science*, Vol. 3 (Ed. J. W. Holmes), pp. 429–435. Adelaide: International Society of Soil Science.

[ref16] Greenwood K. L. & McKenzie B. M. (2001). Grazing effects on soil physical properties and the consequences for pastures: a review. Australian Journal of Experimental Agriculture 41, 1231–1250.

[ref17] Hahn M. V., McDaniel B. T. & Wilk J. C. (1984). Genetic and environmental variation of hoof characteristics of Holstein cattle. Journal of Dairy Science 67, 2986–2998. 6530494 10.3168/jds.S0022-0302(84)81664-1

[ref18] Heins B. J., Hansen L. B., Hazel A. R., Seykora A. J., Johnson D. G. & Linn J. G. (2012). Short communication: Jersey × Holstein crossbreds compared with pure Holsteins for body weight, body condition score, fertility, and survival during the first three lactations. Journal of Dairy Science 95, 4130–4135. 22720969 10.3168/jds.2011-5077

[ref19] Hennessy D., Moran B., Kinsella A. & Quinlan G. (2011) Teagasc National Farm Survey Results 2010. Athenry, Ireland: Teagasc www.teagasc.ie/publications/2011/1016/NFS10.pdf (verified September 2014).

[ref20] Herbin T., Hennessy D., Richards K. G., Piwowarczyk A., Murphy J. J. & Holden N. M. (2011). The effects of dairy cow weight on selected soil physical properties indicative of compaction. Soil Use and Management 27, 36–44.

[ref21] Houlbrooke D. J., Thom E. R., Chapman R. & McLay C. D. A. (1997). A study of the effects of soil bulk density on root and shoot growth of different ryegrass lines. New Zealand Journal of Agricultural Research 40, 429–435.

[ref22] Humphreys J., O'Connell K. & Casey I. A. (2008). Nitrogen flows and balances in four grassland-based systems of dairy production on a clay-loam soil in a moist temperate climate. Grass and Forage Science 63, 467–480.

[ref23] Jarrige R. (1989). Ruminant Nutrition: Recommended Allowances and Feed Tables. Paris, France: John Libbey Eurotext.

[ref24] Jarrige R., Demarquilly C., Dulphy J. P., Hoden A., Robelin J., Beranger C., Geay Y., Journet M., Malterre C., Micol D. & Petit M. (1986). The INRA ‘fill unit’ system for predicting the voluntary intake of forage-based diets in ruminants: a review. Journal of Animal Science 63, 1737–1758.

[ref25] Lipiec J. & Hatano R. (2003). Quantification of compaction effects on soil physical properties and crop growth. Geoderma 116, 107–136.

[ref26] Mapfumo E. & Chanasyk D. S. (1998). Guidelines for safe trafficking and cultivation, and resistance–density–moisture relations of three disturbed soils from Alberta. Soil and Tillage Research 46, 193–202.

[ref27] McDowell R. W. (2008). Environmental Impacts of Pasture Based Farming. Wallingford, UK: CABI.

[ref28] Menneer J. C., Ledgard S. F., McLay C. D. A. & Silvester W. B. (2005). The effects of treading by dairy cows during wet soil conditions on white clover productivity, growth and morphology in a white clover–perennial ryegrass pasture. Grass and Forage Science 60, 46–58.

[ref29] Morgan D. J., Stakelum G., & Dwyer J. (1989). Modified neutral detergent cellulase digestibility procedure for use with the ‘Fibertec’ system. Irish Journal of Agricultural Research 28, 91–92.

[ref30] Mullen G. J., Jelley R. M. & McAleese D. M. (1974). Effects of animal treading on soil properties and pasture production. Irish Journal of Agricultural Research 13, 171–180.

[ref31] Mullins C. E. & Fraser A. (1980). Use of the drop-cone penetrometer on undisturbed and remoulded soils at a range of soil-water tensions. Journal of Soil Science 31, 25–32.

[ref32] Mulqueen J. (1974). Drainage of Impeded Soils. Dublin: An Foras Taluntais.

[ref33] Necpalova M., Fenton O., Casey I. & Humphreys J. (2012). N leaching to groundwater from dairy production involving grazing over the winter on a clay-loam soil. Science of the Total Environment 432, 159–172. 22728303 10.1016/j.scitotenv.2012.05.091

[ref34] Nie Z. N., Ward G. N. & Michael A. T. (2001). Impact of pugging by dairy cows on pastures and indicators of pugging damage to pasture soil in south-western Victoria. Australian Journal of Agricultural Research 52, 37–43.

[ref35] O'Mara F. (1996). A Net Energy System for Cattle and Sheep. Dublin, Ireland: Department of Animal Science, Faculty of Agriculture, University College Dublin.

[ref36] Patton D., Shalloo L., Pierce K. M. & Horan B. (2012). A biological and economic comparison of 2 pasture-based production systems on a wetland drumlin soil in the northern region of Ireland. Journal of Dairy Science 95, 484–495. 22192229 10.3168/jds.2011-4558

[ref37] Phelan P., Keogh B., Casey I. A., Necpalova M. & Humphreys J. (2013 *a*). The effects of treading by dairy cows on soil properties and herbage production for three white clover-based grazing systems on a clay loam soil. Grass and Forage Science 68, 548–563.

[ref38] Phelan P., Casey I. A. & Humphreys J. (2013 *b*). The effect of target postgrazing height on sward clover content, herbage yield, and dairy production from grass-white clover pasture. Journal of Dairy Science 96, 1598–1611. 23332838 10.3168/jds.2012-5936

[ref39] Piwowarczyk A., Giuliani G. & Holden N. M. (2011). Can soil moisture deficit be used to forecast when soils are at high risk of damage owing to grazing animals? Soil Use and Management 27, 255–263.

[ref40] Prendiville R., Pierce K. M. & Buckley F. (2009). An evaluation of production efficiencies among lactating Holstein-Friesian, Jersey, and Jersey × Holstein-Friesian cows at pasture. Journal of Dairy Science 92, 6176–6185. 19923621 10.3168/jds.2009-2292

[ref41] Prendiville R., Lewis E., Pierce K. M. & Buckley F. (2010). Comparative grazing behavior of lactating holstein-friesian, jersey, and jersey × holstein-friesian dairy cows and its association with intake capacity and production efficiency. Journal of Dairy Science 93, 764–774. 20105548 10.3168/jds.2009-2659

[ref42] Saleh A. (1994). Measuring and predicting ridge-orientation effect on soil surface roughness. Soil Science Society of America Journal 58, 1228–1230.

[ref43] SAS Institute (2006). SAS User Guide. Version 9.1. Cary, NC: SAS Institute Inc.

[ref44] Scholefield D. & Hall D. M. (1985). A method to measure the susceptibility of pasture soils to poaching by cattle. Soil Use and Management 1, 134–138.

[ref45] Scholefield D. & Hall D. M. (1986). A recording penetrometer to measure the strength of soil in relation to the stresses exerted by a walking cow. Journal of Soil Science 37, 165–176.

[ref46] Schulte R. P. O., Diamond J., Finkele K., Holden N. M. & Brereton A. J. (2005). Predicting the soil moisture conditions of Irish grasslands. Irish Journal of Agricultural and Food Research 44, 95–110.

[ref47] Shalloo L., Dillon P., O'Loughlin J., Rath M. & Wallace M. (2004). Comparison of a pasture-based system of milk production on a high rainfall, heavy-clay soil with that on a lower rainfall, free-draining soil. Grass and Forage Science 59, 157–168.

[ref48] Smit H. J., Metzger M. J. & Ewert F. (2008). Spatial distribution of grassland productivity and land use in Europe. Agricultural Systems 98, 208–219.

[ref49] Tyrell H. F. & Reid J. T. (1965). Prediction of the energy value of cow's milk. Journal of Dairy Science 48, 1215–1223. 5843077 10.3168/jds.S0022-0302(65)88430-2

[ref50] Vance E. R., Ferris C. P., Elliott C. T., Hartley H. M. & Kilpatrick D. J. (2013). Comparison of the performance of Holstein-Friesian and Jersey × Holstein-Friesian crossbred dairy cows within three contrasting grassland-based systems of milk production. Livestock Science 151, 66–79.

[ref51] Willatt S. T. & Pullar D. M. (1984). Changes in soil physical properties under grazed pastures. Australian Journal of Soil Research 22, 343–348.

[ref52] Xue B., Yan T., Ferris C. F. & Mayne C. S. (2011). Milk production and energy efficiency of Holstein and Jersey-Holstein crossbred dairy cows offered diets containing grass silage. Journal of Dairy Science 94, 1455–1464. 21338810 10.3168/jds.2010-3663

